# Electrical Impedance Myography in Health and Physical Exercise: A Systematic Review and Future Perspectives

**DOI:** 10.3389/fphys.2021.740877

**Published:** 2021-09-14

**Authors:** Álex Cebrián-Ponce, Alfredo Irurtia, Marta Carrasco-Marginet, Gonzalo Saco-Ledo, Montserrat Girabent-Farrés, Jorge Castizo-Olier

**Affiliations:** ^1^Barcelona Sports Sciences Research Group, Institut Nacional d'Educació Física de Catalunya (INEFC), Universitat de Barcelona (UB), Barcelona, Spain; ^2^Bioenergy and Motion Analysis Laboratory, National Research Center on Human Evolution (CENIEH), Burgos, Spain; ^3^Tecnocampus, Universitat Pompeu Fabra, Barcelona, Spain

**Keywords:** muscle, physiology, bioimpedance, bioelectrical vector, sport

## Abstract

**Background:** Electrical impedance myography (EIM) is a non-invasive method that provides information about muscle health and changes that occur within it. EIM is based on the analysis of three impedance variables: resistance, reactance, and the phase angle. This systematic review of the literature provides a deeper insight into the scope and range of applications of EIM in health and physical exercise. The main goal of this work was to systematically review the studies on the applications of EIM in health and physical exercise in order to summarize the current knowledge on this method and outline future perspectives in this growing area, including a proposal for a research agenda. Furthermore, some basic assessment principles are provided.

**Methods:** Systematic literature searches on PubMed, Scopus, SPORTDiscus and Web of Science up to September 2020 were conducted on any empirical investigations using localized bioimpedance devices to perform EIM within health and physical exercise contexts. The search included healthy individuals, elite soccer players with skeletal muscle injury, and subjects with primary sarcopenia. The Preferred Reporting Items for Systematic Reviews and Meta-Analyses (PRISMA) checklist was used to develop the systematic review protocol. The quality and risk of bias of the studies included were assessed with the AQUA tool.

**Results:** Nineteen eligible original articles were included in this review, which were separated into three tables according to the nature of the study. The first table includes six studies on the bioelectrical characterization of muscle. The second table includes five studies analyzing muscle changes in injured elite soccer players. The third table includes studies on the short-, medium-, and long-term bioelectrical adaptations to physical exercise.

**Conclusions:** EIM has been used for the evaluation of the muscle condition in the clinical field over the last few years, especially in different neuromuscular diseases. It can also play an important role in other contexts as an alternative to complex and expensive methods such as magnetic resonance imaging. However, further research is needed. The main step in establishing EIM as a valid tool in the scientific field is to standardize the protocol for performing impedance assessments.

## Introduction

Bioimpedance assessment carried out on a particular part of the human body is known as localized bioimpedance analysis (L-BIA). If the specific area analyzed is a muscle or a group of muscles, it is known either as muscle-localized bioimpedance analysis or electrical impedance myography (EIM), a term that was first introduced in 2002 (Rutkove et al., 2002), but had already been studied before (Elleby et al., 1990). EIM is a non-invasive, painless assessment method that quantifies the passive electrical behavior of muscle (Rutkove, 2009). It involves the application of an electrical current and the measurement of the voltage across the biological tissue. It has been proposed that EIM can provide detailed physiological data on the composition and structure of muscle (Sanchez and Rutkove, 2017b), depending on factors such as cell populations, cell volumes, cell membrane integrity, and intra-/extracellular fluids of the tissue (Freeborn et al., 2020). EIM provides a set of quantitative parameters, usually: resistance (R), the major resistance to the current through intra- and extracellular ionic fluids; reactance (Xc), the additional resistance due to capacitive elements such as cell membranes, tissue interfaces, and non-ionic substances; impedance (Z), the resistance of the tissues to the electric current flow; and the phase angle (PhA), the geometric relationship between R and Xc (Castizo-Olier et al., 2018). This set of parameters might be useful in the assessment of muscle health and other outcomes.

To date, the main purpose of EIM has been to evaluate neuromuscular diseases, more specifically to assess disease severity, progression over time, and response to therapy; however, it has not been used to establish an initial diagnosis (Sanchez and Rutkove, 2017a). EIM is based on the idea that conductivity and permittivity in a diseased muscle are altered (Foster and Schwan, 1989) and these alterations in the muscle can cause changes in the voltage generated. There are several studies showing that a diseased muscle has different impedance values compared to healthy muscle, for instance, in individuals with neurogenic disorders such as amyotrophic lateral sclerosis (Tarulli et al., 2009), spinal muscular atrophy (Rutkove et al., 2010), and radiculopathy (Spieker et al., 2013) or in those with muscular dystrophies such as Duchenne muscular dystrophy (Rutkove et al., 2014) and facioscapulohumeral muscular dystrophy (Statland et al., 2016). In general, a diseased muscle presents significantly decreased R, Xc, and PhA values, mainly due to muscle fiber atrophy, connective tissue accretion, fat infiltration, and edema (Rutkove et al., 2008; Nescolarde et al., 2020). However, depending on the disease, there may be some parameters that are more useful than others. Thus, future investigations should focus on detecting which parameter is more useful in each condition. This article does not go into detail about the applicability of EIM in neuromuscular diseases since there are recent reviews that address this topic (Sanchez and Rutkove, 2017a,b; Rutkove and Sanchez, 2019).

EIM has also been used to investigate age-related changes in the muscle in sarcopenia, skeletal muscle injuries, and adaptations generated by physical exercise within the muscle.

Sarcopenia is a widespread and progressive disease of the skeletal muscle, characterized by decreased muscle strength, muscle mass, and functional ability, which is often directly related to advanced age (Thompson, 2009; Narici and De Boer, 2011). This condition affects ~25% of individuals aged over 70 years and 30–50% of individuals aged over 80 (Baumgartner et al., 1998), but these prevalence rates may change depending on the cut-off points established for the muscle assessments (Masanés et al., 2017). The most common methods to evaluate sarcopenia are a combination of muscle mass and muscle strength measurements, and radiological imaging (Pillard et al., 2011; Cruz-Jentoft and Sayer, 2019). However, strength measurements require subjective elements well-beyond the properties of muscle tissue itself such as motivation or willingness to participate (Kortman et al., 2013), while radiological imaging is very costly, difficult to apply, and with a great invasiveness (Pahor et al., 2009). For this reason, a quick evaluation method of the muscle condition in sarcopenia is needed to facilitate individual patient care and clinical research. Subjects with sarcopenia present a lower number and smaller size of muscle fibers. Thus, differences in the impedance characteristics of the muscle might be expected (Aaron et al., 2006; Rutkove et al., 2008; de-Mateo-Silleras et al., 2018), supporting the potential application of EIM and overcoming the limitations of other methods. Sarcopenia might be classified into two categories. If the primary cause of sarcopenia is aging, it is considered to be “primary sarcopenia.” If there is another factor other than aging, like a systemic disease, it is considered to be “secondary sarcopenia” (Cruz-Jentoft et al., 2019). Other intrinsic factors that can play an important role in the localized bioimpedance analysis are gender (Aaron et al., 2006; Tarulli et al., 2007; Kortman et al., 2013; Mascherini et al., 2017; de-Mateo-Silleras et al., 2018) and body composition (Narayanaswami et al., 2012; Baidya and Ahad, 2016).

A skeletal muscle injury occurs when a force applied to a muscle causes structural damage such as contusions, elongations or strains of different grades, depending on the severity of the injury (Maffulli et al., 2014). These types of injuries are the most common in sports, where they are a major challenge in primary care and sports medicine (Baoge et al., 2012) with regards to their prevention, the determination of the best time for the return to play (RTP) and the reduction of the re-injury rate (Nescolarde et al., 2013). Some longitudinal studies using EIM have been performed with injured elite male soccer players in the last decade (Nescolarde et al., 2013, 2015, 2017, 2020; Francavilla et al., 2015). In general, the impedance values of the injured muscle are decreased and gradually recover over time during the process of healing. This means that EIM might be a practical non-invasive tool for assessing muscle health quality in physical exercise and sports (Sanchez and Rutkove, 2017b).

There are several investigations using bioelectrical impedance analysis (BIA) in physical exercise and sports. However, most of them have used whole-body BIA and there are only a few applying localized bioimpedance analysis (Castizo-Olier et al., 2018). These studies can be separated into three groups according to the classification of Castizo-Olier et al. (2018). The first group includes short-term investigations (<24 h) with the main purpose of analyzing impedance values during the execution of an exercise (Shiffman et al., 2003; Li L. et al., 2016; Fu and Freeborn, 2018; Freeborn and Fu, 2019; Huang et al., 2020), also known as dynamic electrical impedance myography (D-EIM), a term first coined in 2003 (Shiffman et al., 2003). The second group contains medium-term investigations (≥24 h and <7 days) that aim to assess muscle responses in the days following an exercise protocol (Elleby et al., 1990; Freeborn et al., 2020). The third group includes long-term investigations (≥7 days) analyzing localized chronic adaptations to exercise; however, there is only one study inside this category (Mascherini et al., 2015). While short-term analysis with EIM may be useful in understanding what happens inside a muscle when performing different exercise protocols, medium-, and long-term analyses may be useful in evaluating how training affects muscle during recovery in the days after the exercise has been performed. Therefore, this could help in controlling the training load.

EIM is a relatively new tool for muscle assessment and although there are several studies investigating the topic, it is a technology that is far from being considered as consolidated. For this reason, the assessment technique as well as its applicability in health and physical exercise need to be further studied. If EIM wants to reach its full potential, theoretical, engineering, and clinical improvements are needed (Sanchez and Rutkove, 2017a). To date, the main point of interest regarding EIM has been in studies on neuromuscular diseases, but it may have great potential in other areas, for example, in the recognition of injury and its follow-up during recovery until the RTP, as well as in the tracking of the training load based on physiological changes in the muscle without the use of expensive and complex technology that is not accessible to everyone. Therefore, the increase in the number of publications regarding EIM in physical exercise, sports, and health seems justified in order to investigate the applicability of EIM for assessments in real time and in a precise, accurate, reliable, non-invasive, portable, inexpensive, safe, and simple way. In addition, since studies in these fields are still scarce and very heterogeneous, a compilation of current knowledge is needed to suggest a research agenda.

This systematic review aims to summarize the current knowledge on the use of EIM as a diagnostic tool for muscle adaptations in muscle health and physical exercise. Beyond that, this review attempts to outline future perspectives in this field and suggest a research agenda.

Lastly, this review will also report some basic assessment principles, since there is a variety of ways of using EIM and there are certain aspects that must be taken into account before performing the assessment, like the device, materials, electrode materials and arrangement, frequency, and factors that could affect the results (Tarulli et al., 2007; Kortman et al., 2013; Sung et al., 2013; Mascherini et al., 2015, 2017).

## Methods

The Preferred Reporting Items for Systematic Reviews and Meta-Analyses (PRISMA) guidelines were applied to undertake the present review (Moher et al., 2009). The PRISMA checklist was also used to develop the systematic review protocol (Moher et al., 2015). The checklist is reported in Supplementary Table 1.

### Eligibility Criteria

This study reviewed and analyzed both descriptive and analytic empirical studies that used bioimpedance devices to perform assessments in exercise, sports, or health. Only studies where the assessment was performed on the muscle itself were selected. Articles that used EIM in healthy sedentary people, subjects with primary sarcopenia, physically active individuals, and athletes of all levels were eligible for review. The following studies were included: (a) studies with muscle-localized bioimpedance measurements at 50 kHz, discarding those that analyzed wounds or burns or were performed with animals; (b) investigations on muscle bioelectrical and/or physiological properties, discarding those on BIA technology and its reliability and validity, localized bioimpedance techniques, or reproducibility of the measurements; (c) investigations involving healthy subjects, subjects with skeletal muscle injuries or subjects with primary sarcopenia, excluding those involving subjects with any other type of acute or chronic disease, as well as subjects with secondary sarcopenia; (d) studies published in a peer-reviewed journal; and (e) studies published in the English language. No restrictions in terms of country, time frame, or age of the subjects were considered.

### Information Sources

The present review searched the following databases: PubMed, Scopus, SPORTDiscus and Web of Science. The last update was conducted in mid-September 2020.

### Search Strategy

The title, abstract and keyword fields were searched in each of the aforementioned databases using the following search terms and syntax: [(“EIM” OR “electrical impedance”) AND (“muscle” OR “myography”)] OR [(locali^*^ OR “regional”) AND (“bioimpedance” OR “bioelectrical” OR “BIA” OR “BIVA”)]. This syntax was the same for the four databases used. No data restrictions were applied. However, only studies published in the English language were selected.

### Study Records

Records were exported from the electronic databases to a reference management software (Mendeley Desktop 1.19.4) and duplicated references were removed.

After removing the duplicates, the eligible articles were screened by two investigators (ÁC-P and GS-L), with disagreement settled by consensus. An initial screening of the titles and abstracts was performed to check the eligibility criteria. Differences in study eligibility were compared and deviations were discussed with a third investigator (AI) until consensus was reached. When a paper could not be rejected with certainty, it was included in the eligible papers for full-text evaluation. The articles were then assessed for eligibility through a full-text screening and those meeting the established criteria were included in the review. The reference lists of the articles retrieved for inclusion in the review up to this point were searched to identify other relevant investigations. The number of studies meeting the pre-specified inclusion criteria and those excluded and the reasons for their exclusion were recorded and codified. Each selected article was reviewed for information on the (1) bibliographic characteristics (type of publication, authors, year, and journal); (2) aims of the investigation; (3) study design and methodology; (4) sample characteristics (number, population, gender, age, and condition); (5) BIA characteristics (device employed, extrapolated frequency, electrode arrangement, brand of the electrodes used, and their localization); and (6) results, conclusions, and reported limitations. Furthermore, the nomenclature of each study was respected according to their original articles. All this information was organized and compiled into tables.

### Data Extraction and Prioritization

Full texts were reviewed for the following variables: bioelectrical resistance (R, R/h), reactance (Xc, Xc/h), and the PhA of the muscle selected, as well as the electrode placement to assess bioelectrical values. Bioelectrical measures and directly derived parameters were considered the main outcome, independently of study design. Only data at a frequency of 50 kHz from healthy subjects, subjects with muscle injury, or subjects with primary sarcopenia were analyzed. Bioimpedance analyses different from the muscle-localized one were also obviated.

### Risk of Bias Assessment of the Studies Included

The quality of all the included studies was assessed with the AQUA tool (Henry et al., 2017). This tool is designed to assess the quality and reliability of studies by addressing the risk of bias as “high,” “low,” or “unclear,” using five key domains: (1) characterization of the study target and subject; (2) design of the research; (3) characteristics of the methodology; (4) descriptive anatomy; and (5) reporting of outcomes. If a study did not meet the recommended guidelines ascribed to each domain, it was classified as being at “high” risk of bias within the confines of that specific category. If the domain met the recommended guidelines, it was marked as having a “low” risk of bias. “Unclear” was used in the cases of insufficient or vague data. Two of the authors (ÁC-P and GS-L) screened the articles and assessed the risk of bias for each of the five domains. Disagreement was resolved by consensus or third-party adjudication (AI).

## Results

### Results of the Search

**Figure 1:**
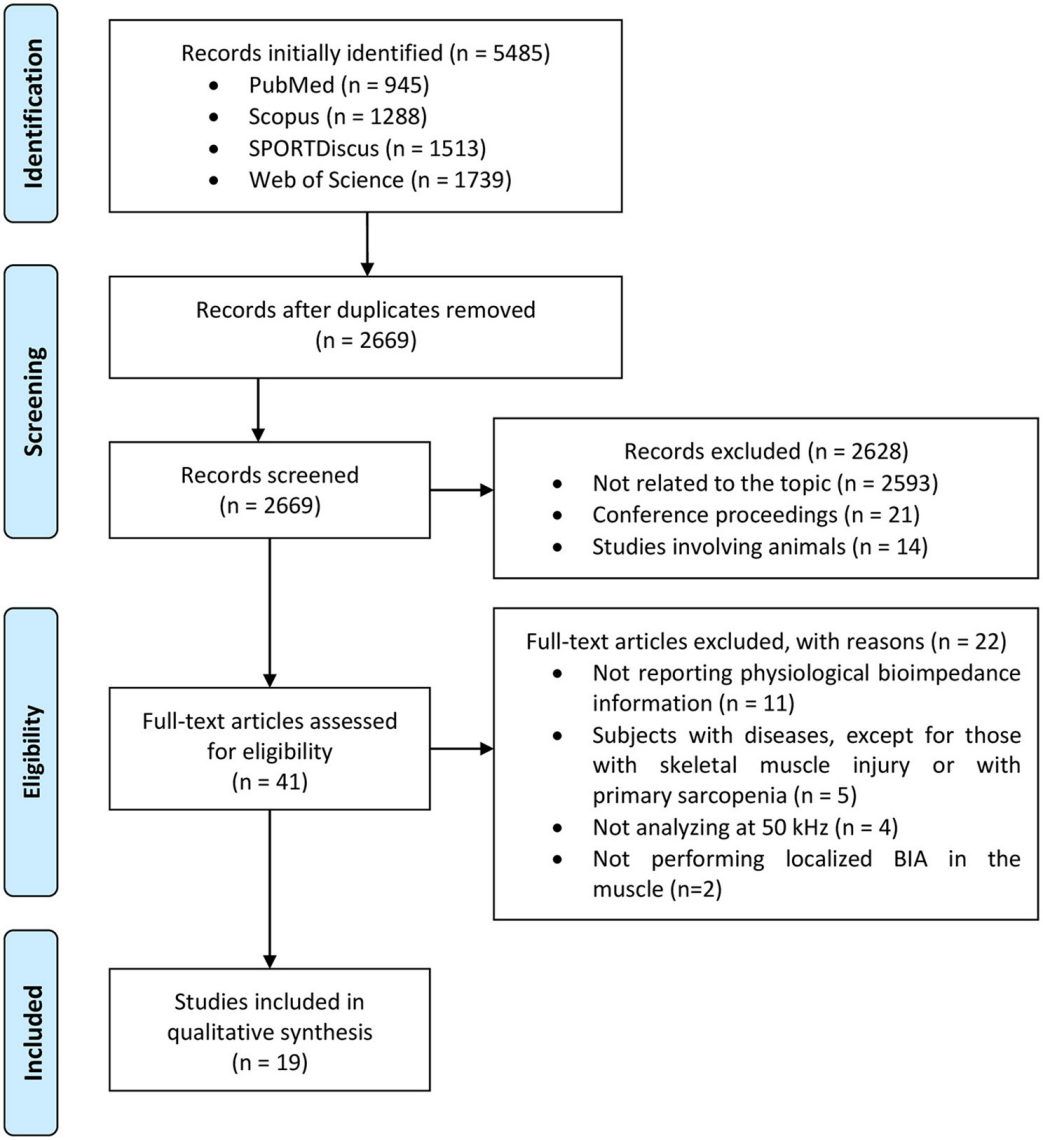
Flow chart of study identification and eligibility for the systematic review.

Figure 1 displays the flow chart of study identification and eligibility for the systematic review. The search term provided 5,485 results, which were reduced to 2,669 after the removal of duplicates. Of the 2,669 records identified, 41 were selected after screening titles, abstracts and keywords, discarding those not related to the topic such as studies on a topic with little or no relationship at all, those not using localized bioimpedance, those involving subjects that are not healthy, or a combination of all of them. Conference proceedings and studies involving animals were also excluded. After full-text evaluation, 19 studies matched the selection criteria and were included in the qualitative synthesis analysis. These are summarized in Tables 1–**3**. The other 22 studies were excluded for different reasons: not reporting physiological bioimpedance information and instead focusing on technological or methodological aspects, not analyzing at 50 kHz, using subjects with diseases, or not performing localized BIA. An important aspect is that the inclusion criteria included studies on healthy subjects, those with skeletal muscle injury and those with primary sarcopenia'. The publication date ranged from 1990 to 2020, but only one study was published before 2003 (Elleby et al., 1990) and only five before 2013. Therefore, the interest in this field of study has increased in the last decade, unlike the studies on neuromuscular diseases that have been studied for longer.

**Table 1:** Reviewed EIM studies on the bioelectrical characterization of muscle.

**Study**	**Aim**	**Design**	**Type of** **analysis**	**Subjects**				**BIA**						**Results**	**Conclusions**	**Reported** **limitations**
				***N***	**Sex**	**Age**	**Condition**	**Device**	**Fr**	**Distribution**	**Localized** **muscle**	**Electrodes** **used**	**Electrode** **arrangement**			
Aaron et al. (2006)	To analyze EIM values in people of different ages	Cross-sectional plus longitudinal measurements up to 4.5 years after the initial assessment of 4 subjects	Intra-group analysis. Comparison between a preliminary study of patients with various ND	100	56 F; 44 M	18–90	Healthy subjects, with data on some of them obtained from a preliminary study	RJL Model 101-A	50 kHz	Localized	Quadriceps, tibialis anterior	019-400500; Viasys Healthcare/ Nicolet Biomedical	Quadriceps: the initial electrode (V1) was placed 10 cm proximal to the patella with V2–V8 placed 2.5 cm apart in a proximal line along the thigh. Tibialis anterior: V6 was placed 2.5 cm distal to the midpoint between the bottom of the patella and the fibular head, with V5–V1 placed 2.5 cm apart in a line down the belly of the muscle along the anterior foreleg	PhA decreased with increasing age, especially after 60 years of age. The correlation was stronger in M (quadriceps: *r^2^* = 0.68 for M, 0.52 for F; tibialis anterior: *r^2^* = 0.74 for M, 0.38 for F; *p* < 0.001 throughout)	Greater reductions in the PhA with increasing age. EIM has potential and is a simple and effort-independent test of muscle health in the elderly	NR
Tarulli et al. (2007)	To determine the impact of SFL thickness on EIM	Cross-sectional	Intra-group and inter-group analysis. Comparison between a wide variety of SFL thicknesses measured by ultrasound	62	29 F; 33 M	52.2 ± 20.6	Healthy	RJL systems BIA-101A	50 kHz	Localized	Quadriceps	019-768500, VIASYS Healthcare/ Nicolet Biomedical and 019-435500, VIASYS Healthcare/ Nicolet Biomedical	I pair: attached to the dorsum of the feet. V pair: over the quadriceps in a standard array with 2.5 cm between the electrodes. Starting from 10 cm proximal to the patella	A significant but weak positive correlation (*r^2^* = 0.14, *p* < 0.05) was seen between age and SFL thickness in F, but not in M. A strong negative correlation between age and PhA was observed for both M (*r^2^* = 0.48, *p* < 0.01) and F (*r^2^* = 0.68, *p* < 0.01). In multiple regression analysis, age but not SFL thickness was found to have a significant association with PhA	SFL did not contribute substantially to the PhA measured by linear-EIM	Some unavoidable inaccuracy in measurements of the SFL thickness with ultrasound. Only healthy subjects were analyzed
Rutkove et al. (2008)	To create a set of EIM reference values in healthy subjects	Cross-sectional	Inter-group analysis. Establishment of reference values	87	46 F; 41 M	52.4 (18.9–90.8)	Healthy	RJL Model 101-A	50 kHz	Localized	Biceps brachii, forearm flexors, quadriceps, tibialis anterior, medial gastrocnemius	019-400500; Viasys Healthcare/ Nicolet Biomedical	For each muscle, the V pair was placed along the long axis of the limb, and the I pair was placed at a distance from this array. For upper extremity muscle measurements, the I pair was placed on the palms of both hands, and for each lower extremity muscle measurements, they were placed on the dorsum of both feet	The reference PhA values for 20, 30, 40, 50, 60, 70 and 80 years, respectively, were: tibialis anterior (7.94, 7.94, 7.75, 7.38, 6.85, 6.2, and 5.47); quadriceps (6.82, 6.81, 6.59, 6.19, 5.64, 4.99, and 4.28); biceps brachii (3.79, 3.88, 3.89, 3.82, 3.68, 3.48, and 3.22); forearm flexors (3.07, 2.99, 2.88, 2.74, 2.58, 2.4, and 2.21); medial gastrocnemius (7.74, 7.74, 7.54, 7.14, 6.58, 5.9, and 5.15)	PhA decreased with age. Results could be altered depending on measurement methodology	NR
Sung et al. (2013)	To identify the EIM parameter least impacted by SFL thickness when using an HEA	Cross-sectional	Inter-group analysis. Comparison between a variety of skin-SFL thicknesses measured by ultrasound	18	9 F; 9 M	50.7 (19–83)	Healthy	Imp SFB7 (HEA)	50 kHz	Localized	Medial gastrocnemius	HEA	Inner electrodes were 3 cm apart and the outer electrodes were 1.5 cm peripheral to that over the center of the muscle belly	For both R and PhA, a strong relationship with subcutaneous fat layer thickness was observed (*r^2^* = 0.64 and *r^2^* = 0.70, respectively, *p* < 0.001 for both). By contrast, for Xc, the relationship was not significant (*r^2^* = 0.07, *p* = 0.30)	Unlike R and PhA, both of which were highly impacted by SFL thickness, the Xc showed no significant relationship	Xc was smaller in magnitude than R, thus potentially making it a noisier measure, which could have contributed to the lack of a relationship with SFL thickness. Possible error measuring SFL thickness
Mascherini et al. (2017)	To compare body composition between sexes using L-BIA in elite soccer athletes	Cross-sectional	Intra-group and inter-group analysis. Comparison between sexes	36	18 F; 18 M	F: 26.2 ± 2.4; M: 26.9 ± 2.5	Healthy elite soccer athletes	BIA-101 ASE, Akern/RJL Systems	50 kHz	Whole-body and localized	Quadriceps, hamstrings, and calves	Ag/AgCl (NR)	Quadriceps: 5 and 10 cm distally from the anterior inferior iliac spine and proximally from the superior pole of the patella. Hamstring: 5 and 10 cm distally from the ischiatic tuberosity and proximal from the popliteal line. Calves: 5 and 10 cm distally from the popliteal line and 15 and 10 cm from the posterior intermalleolar line	Significant differences between (a) F vs. M quadriceps, hamstrings, and calves (*p* = 0.0001); (b) F vs. M quadriceps vs. calves (*p* = 0.0001); (c) F and M hamstrings vs. calves (*p* = 0.0001); and (d) F quadriceps vs. hamstrings (*p* = 0.02). There were no significant differences between the quadriceps and hamstrings in the M sample (*p* = 0.57)	Significant differences in the thighs between the sexes, while in the calves, these differences were reversed for the Xc values	Absence of BIA data for F athletes
de-Mateo-Silleras et al. (2018)	To assess the association between whole-body and calf impedance vectors and muscle mass and strength in a group of elderly individuals	Cross-sectional	Intra-group analysis. Comparison between muscle health, whole-body BIVA and localized BIVA	113	59 F; 53 M	79.8	All types of elderly people	BIA-101 ASE, Akern/RJL Systems	50 kHz	Whole-body and localized	Calves	NR	Two measuring (ES1 and ES2) and two injecting electrodes (EI1 and EI2) were placed on the lateral side of the right leg. The ES1 electrode was placed at the maximum circumference of the calf; ES2 was placed 10 cm distal to ES1. Injecting electrode EI1 was placed 5 cm proximal to ES1, and EI2 was placed 5 cm distal to ES2	Possible associations were analyzed between BIA values obtained using whole-body BIVA and those from localized BIVA in the calf. Although significant correlation coefficients were obtained for all the BIA values in the total sample, the best association was found with R (0.731) (*p* < 0.001). Results were similar in both M and F	Localized BIVA of the calf only distinguished subjects having muscle mass loss, and may be an alternative to conventional BIVA in the assessment of body composition in the elderly	NR

*N, number of subjects; BIA, bioelectrical impedance analysis; BIVA, bioelectrical impedance vector analysis; EIM, electrical impedance myography; F, female; Fr, frequency; HEA, handheld electrode array; I, current injection electrodes; L-BIA, localized bioelectrical impedance analysis; M, males; NR, not reported; PhA, phase angle; R, resistance; SFL, subcutaneous fat layer; V, voltage sensing electrodes; Xc, reactance*.

### Participants and Characteristics of the Studies

A total number of 607 subjects were involved in the studies included in the review, in addition to the unspecified number of participants in the study of Nescolarde et al. (2015), who only indicated 21 muscle injuries. Seven studies were carried out with elite soccer athletes, of which two involved non-injured subjects (Mascherini et al., 2015, 2017) and five involved subjects with skeletal muscle injury (Nescolarde et al., 2013, 2015, 2017, 2020; Francavilla et al., 2015). Despite all the studies including healthy subjects, one of them compared their data with those of a preliminary study in patients with neuromuscular disease in one section of the article (Shiffman et al., 2003), while another included healthy subjects alongside data from healthy control subjects of a previous study focused on neuromuscular disorders, excluding those with disorders (Aaron et al., 2006). No age restrictions were made. All the studies analyzed males and thirteen of them also included females.

The 19 selected studies were classified according to the characteristics of the study objectives. Table 1 is composed of six studies characterizing muscle values according to age (Aaron et al., 2006; Rutkove et al., 2008; de-Mateo-Silleras et al., 2018), gender (Mascherini et al., 2017), or subcutaneous fat layer (SFL) thickness (Tarulli et al., 2007; Sung et al., 2013). Table 2 includes another five studies reporting changes in EIM parameters during injury and recovery in athletes with muscle injuries (Nescolarde et al., 2013, 2015, 2017, 2020; Francavilla et al., 2015). The combination of these two tables may be used to establish some interpretations regarding bioimpedance parameters and muscle health. Table 3 consists of eight studies focusing on changes in EIM values during or after an exercise protocol, with five investigating short-term adaptations (Shiffman et al., 2003; Li L. et al., 2016; Fu and Freeborn, 2018; Freeborn and Fu, 2019; Huang et al., 2020), two studying medium-term changes (Elleby et al., 1990; Freeborn et al., 2020), and one looking at long-term changes (Mascherini et al., 2015). It is worth mentioning that the variability between the studies is large, which makes it difficult to compare them to obtain clear results. For this reason, the studies were classified in such a way that they were as similar as possible.

**Table 2:** EIM studies analyzing skeletal muscle injuries in elite soccer athletes.

**Study**	**Aim**	**Design**	**Type of analysis**	**Subjects**				**BIA**						**Results**	**Conclusions**	**Reported** **limitations**
				***N***	**Sex**	**Age**	**Condition**	**Device**	**Fr**	**Distribution**	**Localized muscle**	**Electrodes used**	**Electrode arrangement**			
Nescolarde et al. (2013)	To report the effects of the severity of muscle injury and recovery in L-BIA variables	Longitudinal. From injury until RTP	Intra-individual analysis. Comparison with MRI results as gold-standard	3	M	21 ± 5	Injured elite soccer athletes (hamstrings and calves)	BIA-101 ASE, Akern/RJL Systems	50 kHz	Localized	Hamstrings and calves	3MRed DotAdult Solid Gel 2239	Calf: 5 cm (I pair) and 10 cm (V pair) distally from the popliteal line and proximal from the posterior intermalleolar line. Proximal hamstrings: 5 cm (V pair) proximally and distally to the point of maximum pain, the I pair adjacent to the others	Compared to non-injury values, R, Xc, and PhA decreased with increasing muscle injury severity: grade I (11.9, 23.5, and 12.1%); grade II (20.6, 31.6, and 13.3%); and grade III (23.1, 45.1, and 27.6%)	R, Xc, and PhA decreased with injury severity. Decreases in R reflected localized fluid accumulation, while reductions in Xc and PhA highlighted disruption of cell membrane integrity and injury	Excessive inter-observer variability
Francavilla et al. (2015)	To report L-BIA changes during the course of muscle injury healing	Longitudinal. From injury until RTP	Intra-individual analysis. Comparison between different measurements after injury with baseline values pre-injury	1	M	24	Injured elite soccer athlete (long head biceps of the leg, grade II)	BIA-101 ASE, Akern/RJL Systems	50 kHz	Localized	Hamstring	Biatrodes, Akern	I pair over the third metatarsal of both feet and the V pair over the muscle	Marked percentage decrease in R, Xc, and PhA over the 12 days following the injury, especially on the 4th day. R: −11.2%; Xc: −52.9%; PhA: −46.5%	R, Xc, and PhA decreased during injury and returned to baseline levels once the injury healed. Decrease in R reflected localized fluid accumulation, while the decrease in Xc and PhA reflected a disruption in cell membrane integrity and cell density during healing	The daily activities of the player posed numerous limits on the routine use of L-BIA
Nescolarde et al. (2015)	To identify the pattern of change in L-BIA values according to injury severity	Cross-sectional. Measurements made 24 h after injury	Intra-individual and intra-group analysis. Comparison with MRI results as gold-standard	21 injuries. 11 grade I, 8 grade II, and 2 grade III. Number of subjects not reported	M	NR	Injured elite soccer athletes (hamstrings and calves)	BIA-101 ASE, Akern/RJL Systems	50 kHz	Localized	Quadriceps, hamstrings, and adductors	Ag/AgCl (COVIDIEN Ref. 31050522)	V pair: 5 cm proximally and distally from the center of the injury. I pair: close to the others. In the vastus intermedius injury (1 case), V pair: 10 cm proximally and distally from the center of the injury. I pair: close to the others. In the adductors, the electrodes were placed in a transverse position, placing the V pair 5 cm medially and laterally from the center of the injury and the I pair close to the others	Compared to non-injury values, R, Xc, and PhA decreased with increasing muscle injury severity: grade I (10.4, 17.5, and 9.0%), grade II (18.4, 32.9, and 16.6%), and grade III (14.1, 52.9, and 43.1%)	R, Xc, and PhA decreased with increasing injury severity. The most significant change was evidenced in Xc 24 h after injury, showing a pattern in line with the severity of the injury. Variations in R were not as indicative	NR
Nescolarde et al. (2017)	To correlate injury severity through L-BIA parameters	Longitudinal. Measurements made 24 h after injury and the day of RTP	Intra-individual and intra-group analysis. Comparison with MRI results as gold-standard	18 (22 muscle injuries)	M	20–28	Injured elite soccer athletes (hamstrings and calves)	BIA-101 ASE, Akern/RJL Systems	50 kHz	Localized	Quadriceps, hamstrings, adductors, and calves	Ag/AgCl (COVIDIEN Ref. 31050522)	V pair: 5 cm proximally and distally from the center of the injury. I pair: close to the others. In the vastus intermedius, the V pair was placed 10 cm proximally and distally to the center of the injury and the I pair close to the others	Compared to non-injury values, R, Xc, and PhA decreased with increasing muscle injury severity: grade I (10.2, 13.4, and 3.2%), grade II with no gap (12.8, 23.5, and 11.2%), and grade II with gap (19.9, 37.5, and 20.5%).	After grouping the data according to the muscle gap (by MRI examination), there were significant differences in R between grade I and grade II with gap, as well as between grade II with gap and grade II with no gap	NR
Nescolarde et al. (2020)	To evaluate by L-BIA different muscle and tendinous injuries 24 h after injury and at their return to play	Cross-sectional. 24 h after injury	Intra-individual and intra-group analysis. Comparison with MRI results as gold-standard	32 (37 muscle injuries)	M	23.5 ± 1.5	Injured elite soccer athletes (hamstrings and calves)	BIA-101 ASE, Akern/RJL Systems (P-S)	50 kHz	Localized	NR	Ag/AgCl (COVIDIEN Ref. 31050522)	V pair: 5 cm proximally and distally from the center of the injury. I pair: close to the others. In injuries located near the bone detector, the V pair was placed 10 cm proximally and distally from the center of the injury, and the I pair close to the others	Significant decrease (*p* < 0.01) in R, Xc, and PhA in both MTJ and MFJ as well as in the different grades of MTJ injuries, particularly in Xc (*p* < 0.001). Regarding days to RTP, there was statistically significant differences among the three different grades of MTJ injuries (*p* < 0.001), especially when grade 1 was compared to grade 3 and grade 2 compared to 3	L-BIA was a complementary method to imaging diagnostic techniques, such as MRI, in the quantification of MTJ and MFJ injuries. In addition, the increase in the severity of the MTJ injury resulted in larger changes in the Xc parameter and a longer time to RTP	To validate L-BIA as a complementary method to MRI, the study should have extended to other professional football teams to increase the sample size and also should have included female athletes

*N, number of subjects; BIA, bioelectrical impedance analysis; EIM, electrical impedance myography; Fr, frequency; I, current injection electrodes; L-BIA, localized bioelectrical impedance analysis; M, males; MFJ, myofascial junction; MRI, magnetic resonance imaging; MTJ, myotendinous junction; NR, not reported; PhA, phase angle; R, resistance; RTP, return to play; V, voltage sensing electrodes; Xc, reactance*.

**Table 3:** EIM studies analyzing adaptations to physical exercise.

**Study**	**Aim**	**Design**	**Type of analysis**	**Subjects**				**BIA**						**Results**	**Conclusions**	**Reported** **limitations**
				***N***	**Sex**	**Age**	**Condition**	**Device**	**Fr**	**Distribution**	**Localized muscle**	**Electrodes used**	**Electrode arrangement**			
Elleby et al. (1990)	To analyze if localized impedance measure-ments could be used to show muscle changes following prolonged intensive exercise	Longitudinal. Medium-term adaptations. Pre-, intra-, post-race (21 km)	Intra-individual analysis. Comparison between measurements that were made for 5 days before the race, immediately after the race, 2 and 6 h after the race, and then daily for 5 days	4	1 F; 3 M	27–47	Healthy	NR	50 kHz	Localized	Calf and thigh	NR	Center of measurement electrodes 17 cm above the patella for the thigh and 17 cm under the patella for the calf. Potential gradients around the thigh/calf	No statistically significant changes in longitudinal ρ measurements. Transverse ρ measurements showed statistically significant changes on the day of the race, but the changes were small. ρ rose during the 6 h following the race. The mean change was 7.0%	Bioimpedance parameters changed following strenuous exercise. However, these changes were small	NR
Shiffman et al. (2003)	To perform EIM during an isometric contraction	Longitudinal. Short-term adaptations	Intra-individual and inter-group analysis. Comparison with a preliminary study of patients with various ND	6	3 F; 3 M	19–70	Healthy individuals plus subjects with disorders obtained from a preliminary study	RJL systems BIA-101A	50 kHz	Localized	Anterior forearm	NR	V pair: 6 cm apart on the anterior forearm, starting about 3 cm from the middle of the elbow joint; I pair: on the abductor pollicis brevis and biceps brachii	Clear relationship between an increase in R and Xc during an isometric contraction, regardless of the force being generated abruptly or gradually	R and Xc increased under MVIC of the finger flexor muscles, changing many milliseconds before the force was generated. After relaxation, R decreased to below baseline levels, while Xc decreased to above baseline levels	NR
Mascherini et al. (2015)	To report changes in localized BIVA after performing a soccer training program	Longitudinal. Long-term adaptations. 50 days between measurements	Intra-group analysis. Comparison between values from pre-season and from the first day of the championships	59	M	22.5 ± 5.6	Healthy elite soccer athlete	BIA-101 ASE, Akern/RJL Systems	50 kHz	Whole-body and localized	Quadriceps, hamstrings, and calves	Biatrodes, Akern	Quadriceps: 5 and 10 cm distally from the anterior inferior iliac spine and proximally from the superior pole of the patella. Hamstrings: 5 and 10 cm distally from the ischiatic tuberosity and proximal from the popliteal line. Calves: 5 and 10 cm distally from the popliteal line and 15 and 10 cm from the posterior intermalleolar line	Values of R/L decreased significantly (hamstrings, 118.01 ± 19.63 vs. 114.49 ± 15.96 Ω/m; quadriceps, 120.80 ± 16.84 vs. 114.71 ± 15.79 Ω/m; calf, 209.57 ± 27.67 vs. 199.72 ± 25.58 Ω/m). There was a greater reduction of R/L in the quadriceps than the hamstrings. This was reflected in an increase in PhA in the quadriceps (13.11 ± 2.13 vs. 13.68 ± 2.25°). However, the greatest muscle modifications were in the calves. Indeed, Xc/L also decreased significantly (50.57 ± 7.39 vs. 48.51 ± 6.97 Ω/m)	The fat did not undergo quantitative changes, but only a change in distribution. Lower limb muscles showed an increase in the amount of water that was more significant than in the whole body assessment. The assessment of body segments under more stress by physical training provided more specific information than the whole body assessment in athletes	Data not valid for non-elite athletes
Li L. et al. (2016)	To assess changes in EIM at different levels of MVIC	Longitudinal. Short-term adaptations	Intra-individual and inter-group analysis. Comparison between different levels of MVIC	19	11 F; 8 M	35 ± 10	Healthy	EIM1103 System, Skulpt (HEA)	50, 100 kHz	Localized	Biceps brachii	HEA	Longitudinal with the muscle fibers over the center of the muscle belly	R increased 10.1 and 9.2% at the high-level contractions (60% MVIC and MVIC). Significant differences in R between the lower contraction (20% MVIC) and higher contraction levels (5.5% increase at 60% MVIC and 4.6% increase at MVIC). No significant changes in R between rest and lower contraction level (20% MVIC). In contrast to R, there were no significant changes in Xc during isometric contraction. For the fatigue effects, R changed significantly during the four different testing conditions	R significantly increased at higher contractions (10% of the baseline value), in contrast to Xc. R also changed during different stages of the fatigue task and there were significant decreases from the beginning of the contraction to task failure as well as between task failure and post-fatigue rest. These changes might be related to the modest alterations in muscle architecture during a contraction	NR
Fu and Freeborn (2018)	To investigate changes in EIM during a fatiguing isotonic protocol	Longitudinal. Short-term adaptations	Intra-individual, intra-group and inter-group analysis. Comparison between two different protocols of bicep curl repetitions at either 60 or 75% 1RM until failure	18	3 F; 15 M	22.2 ± 3.2	Healthy	Keysight E4990A	10, 50, and 100 kHz	Localized	Biceps brachii	Ag/AgCl (Kendall 133)	I pair on the lateralis side of the bicep, 14 cm apart, with the V pair placed 4.67 cm apart	There were significant differences (*p* < 0.05) between the pre- and post-fatigue R and Xc within groups due to the exercise protocol. There were no significant differences (*p* < 0.05) in the R and Xc between the groups at different intensities and no significant differences (*p* < 0.05) of changes between the left and right biceps in both groups	The level of exercise intensity did not significantly affect the changes in electrical impedance of the biceps tissue between the two intensity groups, which is important in monitoring changes as a result of fatigue and exercise-related injury	May be influenced by the subjective reporting of fatigue
Freeborn and Fu (2019)	To investigate EIM changes throughout a fatiguing exercise protocol	Longitudinal. Short-term adaptations	Intra-individual, intra-group and inter-group analysis. Comparison between two different protocols of 10 sets of bicep curl repetitions at either 60 or 75% 1RM	17	3 F; 14 M	22.2 ± 3.2	Healthy	Keysight E4990A	10, 50, and 100 kHz	Localized	Biceps brachii	Ag/AgCl (Kendall 133)	I pair on the lateralis side of the bicep, 14 cm apart, with the V pair placed 4.67 cm apart	At 50 kHz, R decreased 5.6 and 6.6% in the 60% 1RM group, and 5.8 and 6.3% in the 75% 1RM group. Similar trends were observed at 50 and 100 kHz for Xc and PhA measurements of both groups, but this was not as clear	Significant decrease in R, Xc, and PhA compared to the pre-fatigue measures, with the significant R changes occurring at earlier timepoints than Xc and PhA. These results indicated that the electrical impedance of tissues may be an effective monitor in quantifying the changes that occur in localized tissues, including muscle swelling and damage	Not directly measuring the swelling, important in quantifying the relationship with R
Freeborn et al. (2020)	To compare EIM changes throughout a fatiguing exercise protocol	Longitudinal. Medium-term adaptations. Measurements were made over a 96-h observation period post-exercise	Intra-individual, intra-group analysis. Comparison between pre-post protocol involving 50 eccentric repetitions of a dumbbell bicep curl at 90% of their 1RM concentric weight	6	5 F; 1 M	20–25	Healthy	Keysight E4990A	10, 50, and 100 kHz	Localized	Biceps brachii	Ag/AgCl (Kendall 133)	I pair over the biceps brachii 15 cm apart with the V pair placed 6 cm apart. Largest circumference radius of the biceps was the midpoint	Statistically significant differences between the pre-exercise and 72-h (*p* < 0.001) and 96 h post-exercise (*p* < 0.001) *R*-values at 50 kHz. Significant differences between the pre-exercise and 72-h (*p* < 0.001) and 96-h post-exercise (*p* < 0.001) Xc values at 50 kHz	Eccentric protocol increased bicep circumference and decreased magnitude of R and Xc, with maximum changes of swelling and impedance occurring at the same post-protocol timepoints. These changes indicated that EIM is sensitive to the changes within the muscle with the potential to quantify exercise-induced changes	The self-reported pain was only collected for the exercised bicep. No direct measure of tissue damage was obtained
Huang et al. (2020)	To evaluate local muscle fatigue status via changes in EIM	Short-term adaptations	Intra-individual analysis. Comparison between dynamic contraction EIM values at different intensities	8	4 F; 4 M	NR	Healthy	Rigol DG4162 signal generator; Agilent 1141A differential probe, and Agilent MSO7054A oscilloscope	1 kHz−1 MHz. Focus on 50 kHz	Localized	Biceps brachii	Physiotherapy electrodes, 40 × 10 mm–Shanghai Litu Medical Equipment Co., Ltd	Symmetrically along the direction of the arm with the biceps brachii muscle belly as the center. Electrode configuration optimized via a finite element model	R decreased by almost 8 Ω from the completely relaxed muscle to exhaustion, which was the same trend as that for the median frequency parameter of surface electromyography	Subjects were considered to reach the semi-fatigue point when R decreased by approximately 4 Ω. With the decrease in R approaching 8 Ω, muscle fatigue reached its limit, decreasing more quickly with heavier loads. Relationship with a decrease of the median frequency obtained via sEMG	Only evaluated R, not Xc nor PhA

*N, number of subjects; BIA, bioelectrical impedance analysis; BIVA, bioelectrical impedance vector analysis; BMI, body mass index; EIM, electrical impedance myography; F, female; Fr, frequency; HEA, handheld electrode array; I, current injection electrodes; L, length; M, males; MVIC, maximum voluntary isometric contraction; ND, neuromuscular diseases; NR, not reported; PhA, phase angle; R, resistance; RM, repetition maximum; sEMG, surface electromyography; V, voltage sensing electrodes; Xc, reactance; ρ, resistivity*.

### Bioelectrical Measures

All the studies performed localized bioimpedance measurements. However, three of them also included whole-body electrode distribution (Mascherini et al., 2015, 2017; de-Mateo-Silleras et al., 2018). Thirteen studies analyzed the muscles of the lower body and seven studies assessed the muscles of the upper body. All the studies involving subjects with muscle injuries performed the assessments in the lower body. Thirteen investigations used single-frequency impedance devices (50 kHz). The other six studies used multi-frequency bioimpedance analyzers, but also focusing on 50 kHz (Sung et al., 2013; Li L. et al., 2016; Fu and Freeborn, 2018; Freeborn and Fu, 2019; Freeborn et al., 2020; Huang et al., 2020). Furthermore, two of these studies used a handheld electrode array (HEA) to measure bioelectrical values (Sung et al., 2013; Li L. et al., 2016).

### Quality Assessment of the Studies Included

**Figure 2:**
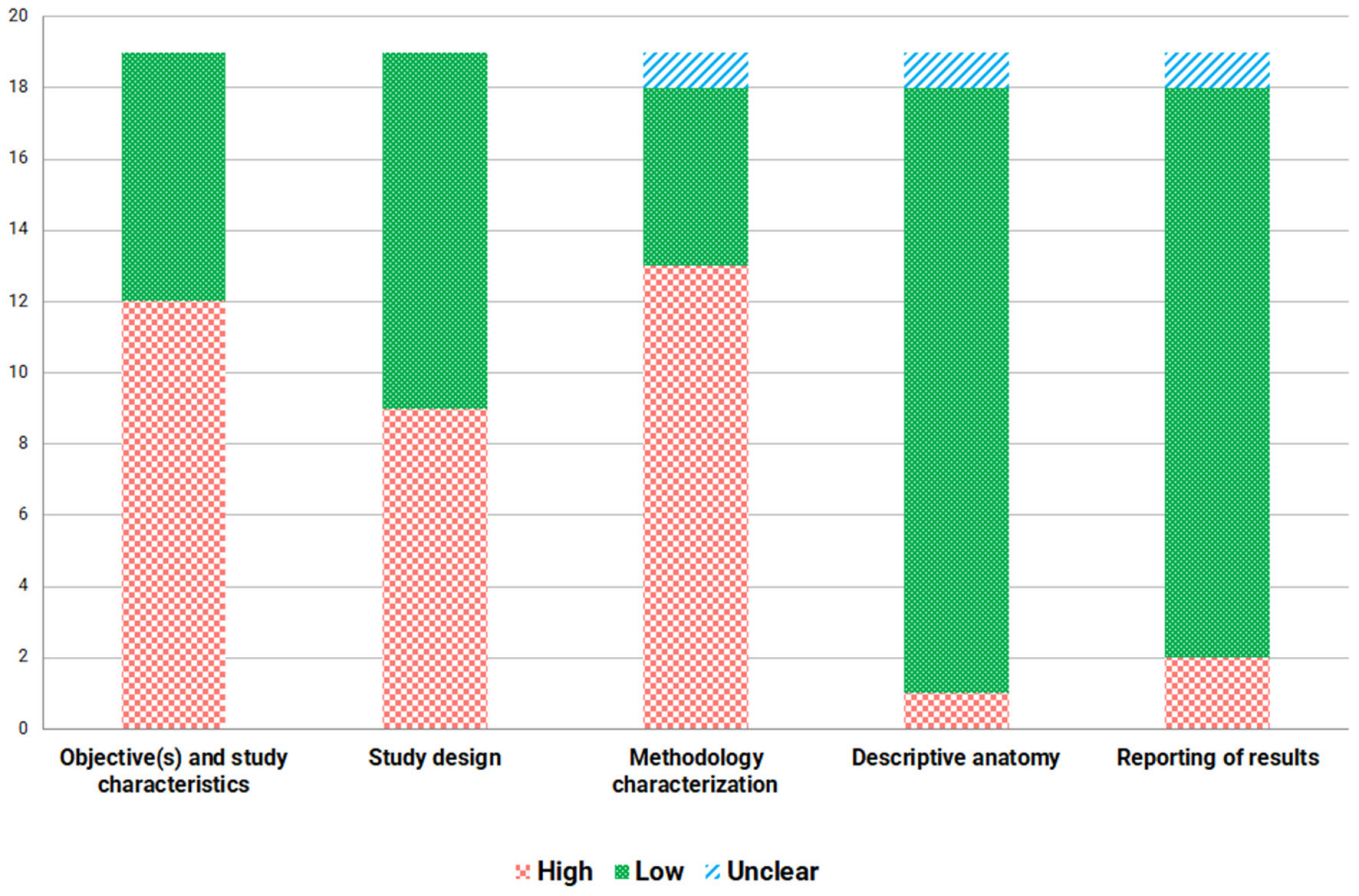
Diagram of the quality assessment of the studies included.

A quality assessment of the studies is presented in Table 4 and Figure 2. The majority of the studies included in this review presented a “high” risk of bias in three domains. The high risk of bias in the objectives and study characteristics domain was mainly due to the small sample size. To determine the risk in this domain, we first focused on whether the authors carried out a study with the ideal sample size and if they complied with it. If not, we established a size of at least 30 subjects for the study to be considered as having a “low” risk of bias, since this sample size is considered large enough to assume normality in the absence of a power analysis (Pandis, 2015). The second domain, study design, also presented a “high” risk of bias in more than half of the studies due to the differences in the placement of the electrodes, since we considered that the appropriate way to perform EIM is through 4 electrodes placed correctly on a specific muscle as this is the most common electrode configuration (Rutkove, 2009). Any other method, such as an HEA or linear-EIM (array of voltage electrodes), was considered as having a “high” risk of bias when establishing appropriate comparisons using the same methodology. The methodology characterization domain was the most affected by a “high” risk of bias, since there was almost no information on the measures taken to reduce inter- and intra-observer variability. The fourth and fifth domains presented a clear “low” risk of bias as the anatomical descriptions of the muscles measured were appropriate, as well as the statistical results. It should be noted that there has been an improvement in the quality of the studies over the years.

**Table 4:** Quality assessment of the included studies with the AQUA tool.

**Studies**	**Risk of bias**				
	**Objective(s) and study characteristics**	**Study design**	**Methodology characterization**	**Descriptive anatomy**	**Reporting of results**
Elleby et al. (1990)	High	High	High	High	High
Shiffman et al. (2003)	High	High	Low	Low	Unclear
Aaron et al. (2006)	Low	High	High	Low	Low
Tarulli et al. (2007)	Low	High	High	Low	Low
Rutkove et al. (2008)	Low	High	High	Low	Low
Nescolarde et al. (2013)	High	Low	High	Low	Low
Sung et al. (2013)	High	High	High	Low	Low
Francavilla et al. (2015)	High	High	High	Low	Low
Mascherini et al. (2015)	Low	Low	High	Low	Low
Nescolarde et al. (2015)	High	Low	Low	Low	Low
Li L. et al. (2016)	High	High	High	Low	Low
Mascherini et al. (2017)	Low	High	High	Low	Low
Nescolarde et al. (2017)	High	Low	Low	Low	Low
de-Mateo-Silleras et al. (2018)	Low	Low	High	Low	High
Fu and Freeborn (2018)	High	Low	Low	Low	Low
Freeborn and Fu (2019)	High	Low	High	Low	Low
Freeborn et al. (2020)	High	Low	Unclear	Low	Low
Huang et al. (2020)	High	Low	High	Low	Low
Nescolarde et al. (2020)	Low	Low	Low	Unclear	Low

## Discussion

Despite EIM being mainly applied in the evaluation of neuromuscular disorders, we discuss its other applications of interest related to health and physical exercise in this review.

We consider that this technique still has much scope for improvement for use as a reference. However, what is undeniable is that it does have the capacity to assess muscle health, providing a quantitative index of the muscle condition (Rutkove and Sanchez, 2019). However, it is still not clear how to interpret the results in all the cases. The great advantages that this technology presents over others make it worthwhile to continue researching so that it can be accessible to everyone.

### Muscle Characterization

The impedance values of the muscle may change according to intrinsic factors such as age, gender or the skin-SFL thickness.

Aaron et al. (2006) found a reduction in PhA values with increasing age, which declined more sharply after 60 years. Despite these changes being significant for both genders, the correlation between the decreased PhA and age was more remarkable in men (quadriceps: *r*^2^ = 0.68 for men and 0.52 for women; tibialis anterior: *r*^2^ = 0.74 for men and 0.38 for women; *p* < 0.001). In the same study, four subjects aged over 75 years repeated the test over a minimum of 3 years. Although the decline was not linear, the slope was between −0.3 and −0.6° per year, exhibiting the age dependence of the PhA. This is an important factor for EIM to measure inherent muscle qualities, avoiding any difficulties associated with contraction and central activation, which is especially of interest in studies on the elderly. It should be noted that the study used a linear electrode array arrangement instead of the classic tetrapolar electrode placement. However, this method also presents excellent test-retest reproducibility (Rutkove et al., 2006). The study did not report the R and Xc values.

Rutkove et al. (2008), to create a set of normal reference values for the PhA of different muscles (the biceps brachii, forearm flexors, quadriceps, tibialis anterior, and medial gastrocnemius), analyzed 87 healthy subjects aged 18.9 to 90.8 years. Again, a clear relationship was found between the PhA and age, with the PhA decreasing with increasing age in all five groups of muscles. The decrease of the PhA seemed to be more pronounced in the muscles of the lower extremities, which is consistent with the fact that loss of muscle mass and strength due to age (primary sarcopenia) occurs especially in the lower body (Janssen et al., 2000). The R and Xc data were not provided. Regarding the electrode arrangement, a pair of voltage electrodes was placed along the long axis of the limb of the specific muscle, while a pair of current electrodes was placed on the dorsum of the foot or palm of the hand regardless of whether the muscle was of the upper or lower body. Therefore, not all the electrodes were located over the muscles of interest.

Janssen et al. (2002) and de-Mateo-Silleras et al. (2018) established a relationship with tolerance ellipses between body composition (through whole-body BIA) and loss of muscle mass and strength in elderly subjects. Localized bioelectrical impedance vector analysis (BIVA) of the calf muscles only distinguishes subjects with loss of muscle mass. Therefore, by using whole-body BIA, de-Mateo-Silleras et al. (2018) could not correlate with a reduced maximum grip strength, which is considered a useful marker in monitoring functional autonomy and the risk of falls (Hairi et al., 2010; Sallinen et al., 2010). Localized BIVA might be an alternative to BIA/BIVA in the cases where the latter techniques cannot be performed for some reason (e.g., prothesis, pacemakers, individuals with edemas, or dehydration).

Regarding gender, Mascherini et al. (2017) showed significant differences in the lower limbs between athletes of the same age and competitive level through localized BIVA. Males presented significantly lower *R*-values than females in the quadriceps (107.6 ± 11.9 vs. 134.4 ± 15.0, *p* = 0.0001), hamstrings (104.9 ± 11.4 vs. 118.8 ± 16.4, *p* = 0.006), and calves (182.1 ± 18.2 vs. 225.9 ± 27.7, *p* = 0.0001). They also showed significantly higher Xc and PhA values in all three muscles, except for the calves, which were higher in females.

As previously mentioned, the presence of fat is a major aspect to consider when measuring impedance parameters. Baidya and Ahad (2016) observed reductions in the R and Xc values with increasing muscle thickness, and increases in the R and Xc values with increasing SFL thickness. Fat tends to increase with increasing age, even in healthy elderly adults (Bai et al., 2018), resulting in changes in the EIM variables. Therefore, this should be taken into consideration when performing assessments. Tarulli et al. (2007) used ultrasound to evaluate SFL thickness in 62 healthy subjects, finding a weak positive correlation between age and SFL thickness in women (*r*^2^ = 0.14, *p* < 0.05) but not in men, as well as a strong negative correlation between age and the PhA for both men (*r*^2^ = 0.48, *p* < 0.01) and women (*r*^2^ = 0.68, *p* < 0.01). However, no significant association was found between the PhA and SFL thickness. As in the study of Aaron et al. (2006), the measurement was not performed using the tetrapolar arrangement, but with linear-EIM, placing eight voltage electrodes in a standard array over the quadriceps, while the current electrodes were attached to the dorsum of the feet. However, the measurement of SFL thickness with ultrasound was made only between the voltage electrodes four and five rather than the entire array, which may vary along the length of the muscle. Furthermore, no data on the R and Xc values were provided.

By contrast, Sung et al. (2013) showed a great impact of SFL thickness on both the R (*r*^2^ = 0.64; *p* < 0.001) and PhA values (*r*^2^ = 0.70; *p* < 0.001), but not on Xc values (*r*^2^ = 0.07; *p* = 0.30), which remained unaltered. However, the major difference regarding the previous study was the assessment technique, as Sung et al. (2013) used an HEA to measure the EIM parameters, unlike Tarulli et al. (2007), who used linear-EIM. An HEA may be less sensitive because the current penetrates only the superficial area of the muscle (Jafarpoor et al., 2013; Rutkove et al., 2017). We need to consider that the current will always take the path of least resistance and if the electrodes are close, the measured voltages will be influenced by the SFL thickness, leading to the evaluation of a mix of muscle and fat. Furthermore, the electrode arrangement of an HEA is always the same, with no variation in the distance between the inner and outer electrodes, which is a relevant point. The correct placement of the electrodes along the muscle belly is important, since small alterations may imply very significant modifications of the results (Jafarpoor et al., 2013; Sanchez et al., 2016b; Rutkove et al., 2017), making little sense to use the same distribution for small and large muscles. For that reason, it is important to highlight the need for choosing the appropriate electrical impedance assessment method, taking care when comparing studies with different techniques since the results may change considerably from one method to another. In this case, an HEA could lead to errors in subjects with higher SFL thickness, as Rutkove et al. (2017) indicated. Baidya and Ahad (2016) also suggested that the injection electrodes should be at the far end of the muscle group, while sensor electrodes should be closer to each other to eliminate variations caused by SFL or muscle thickness.

Taking all these points into consideration, we clearly see that the impedance parameters are altered by factors such as age, gender, fat, and the muscle area analyzed. Furthermore, EIM can be used to assess muscle health beyond neuromuscular diseases. However, the main problem in this section is the great inconsistency between the studies when it comes to performing assessments due to the use of very different techniques, making it very difficult to compare them and make relevant conclusions.

### Injury Follow-Up

There is a series of studies that deal with the impedance changes occurring in a muscle when it gets injured. Considering that localized bioimpedance analysis can assess soft tissue hydration and cell membrane integrity, it is to be expected that some impedance parameters may change when a muscle injury occurs, as well as during the whole process of healing, due to changes in the distribution of fluids and in the cell structure (Järvinen et al., 2005).

Nescolarde et al. (2013) was the first to study this topic, performing tetrapolar measurements of the quadriceps, hamstrings, and calves in a soccer team during one season. During the season, three injuries of different severity (grade I, grade II, and grade III) were analyzed and classified with magnetic resonance imaging (MRI). They found that the R, Xc, and PhA values decreased with increasing severity of the injury and gradually recovered over time as the injury healed. The percentage of decrease in the R, Xc, and PhA values were −23.1, −45.1, and −27.6%, respectively, in the grade III injury and −11.9, −23.5, and −12.1%, respectively, in the grade I injury with regards to the contralateral non-injured side. This pattern of changes during recovery was similar to that obtained with the L-BIA of wound healing (Lukaski and Moore, 2012). The authors indicated that the changes in the *R*-values were due to fluid accumulation in the injured area, while the decreases in the X_C_ and PhA values were the result of disruption to cell membrane integrity and injury. Despite the fact that the sample size was too small for it to be significant, a clear relationship between the decrease in bioelectrical values and injury was seen that would merit further investigation.

Following the same protocol, Nescolarde et al. (2015) built on their previous study (Nescolarde et al., 2013), showing very similar results, but this time the most significant change was evidenced in the Xc value 24 h after injury (*p* < 0.001), with a decrease of −17.5% in grade I injuries (eleven injuries), −32.9% in grade II injuries (eight injuries), and −52.9% in grade III injuries (three injuries). This pattern was in line with the severity of the injury, while variations in the R (−10.4, −18.4, and −14.1% for grade I, II, and III injuries, respectively), and PhA (−9, −16.6, and −43.1% for grade I, II, and III injuries, respectively), were not as indicative. The major difference from their previous study was a larger sample size, although it was not very large, which provided more statistically significant data. A limitation of the study was that the total number of subjects was not reported, with the total number of injuries provided instead. There was a possibility that one player could have suffered more than one injury, which we cannot know.

In a case report of a soccer player with a grade II injury (proximal tendon of the long head of the femoral biceps) from Francavilla et al. (2015), serial measurements were performed until the RTP. Despite the electrode array being different to that of previous studies (Nescolarde et al., 2013, 2015) given that the current injection electrodes were placed on the feet and not on the muscle of interest, the results were very similar 24 h post-injury. However, the greatest changes were observed on the fourth day post-injury, when the PhA and Xc values reached −51.2 and −47.1%, respectively. After that, both substantially increased until the RTP (day 39 post-injury), reaching values similar to those of non-injured players, except for the *R*-values. Both Nescolarde et al. (2015) and Francavilla et al. (2015) agreed that the R is not as indicative as the other parameters since it seems to be strongly influenced by the position of the subject and physical activities. Furthermore, according to previous studies using MRI criteria (Schneider-Kolsky et al., 2006; Reurink et al., 2014), the authors indicated that the presence of edema in the injured muscle did not correlate with the grade of the injury. Thus, R is the least suitable parameter in the management of a muscle injury, unlike the Xc and PhA.

Valle et al. (2017) proposed a new classification of muscle injuries in which the muscle gap plays an important role as an indicator of injury severity. However, this classification does not always allow the identification of injuries by MRI. Nescolarde et al. (2017) proposed L-BIA using tetrapolar assessment to overcome this limitation, since they found that the severity of the injury correlated with the muscle gap according to the RTP values. They noted that a greater injury severity resulted in larger decreases in the R, Xc, and PhA, whereas the presence and severity of muscle disruption adversely affected the time to RTP (*p* < 0.0001). In agreement with previous studies, the authors indicated that L-BIA can accurately predict the time to RTP according to the impedance values of the non-injured limb.

Based on another type of classification of muscle injuries (Pollock et al., 2016) and considering the histoarchitectural approach to skeletal muscle injury (Balius et al., 2020), Nescolarde et al. (2020) classified 37 muscle injuries into 4 different grades based on the severity and nature of the injury (tendinous, myotendinous junction, or myofascial junction injuries), using MRI and ultrasound assessment. They made a cross-sectional analysis measuring injured subjects through the tetrapolar technique. Again, significant decreases (*p* < 0.01) in the R, Xc, and PhA values were found 24 h after injury in both the myotendinous junction (−10.7 ± 6.3, −20 ± 10.1, and −9.9 ± 7, respectively), and myofascial junction (−18.7 ± 5.2, −33.7 ± 8.4, and −17 ± 8.4, respectively), as well as in the different grades of myotendinous junction injuries. The greater changes were observed in the Xc (*p* < 0.001). Regarding the days to RTP, there was a statistically significant difference among the three different grades of myotendinous junction injuries (*p* < 0.001). Therefore, L-BIA is a useful technique for assessing muscle injuries.

One aspect to highlight is that in contrast to the previous section on muscle characterization, almost all the studies on injury, except for that of Francavilla et al. (2015), worked with very similar protocols involving the same authors. They used subjects with similar characteristics, the same devices and materials, the same assessment method, and the same electrode placement, with the protocol improved each time. This emphasizes the importance of standardizing protocols for future research on different EIM applications in order to make more reliable comparisons between studies and to move forward with this technique.

In conclusion, EIM is a practical method for characterizing the severity of muscle injuries and monitoring follow-up until the RTP. Xc and PhA values abruptly decrease when an injury occurs, increasing linearly and consistently during the healing phase. In these studies, different classifications of muscle injuries were used and in all of them, EIM was able to identify the severity of the injury. One of the major problems in sports medicine is reinjury (Orchard and Best, 2002). EIM could play an important role in minimizing its risk in athletes or sport entities that cannot afford alternative and more expensive methods like MRI and ultrasound (Rutkove, 2020). However, more research is needed, especially studies with larger sample sizes, investigations analyzing different sports, and longitudinal studies monitoring the follow-up from injury to full recovery.

### Adaptations Generated by Physical Exercise

Acute and chronic physical exercise induces some morphological and physiological adaptations within the muscle and these changes in muscle properties can be quantified by EIM. There are some studies analyzing this topic and they can be classified into three groups based on what they are investigating: (1) short-term adaptations; (2) medium-term adaptations; and (3) long-term adaptations.

### Short-Term Adaptations

The majority of the studies included in this review investigated the short-term adaptations to physical exercise. The first study using D-EIM in healthy subjects by Shiffman et al. (2003) assessed the anterior forearm muscles using a tetrapolar electrode arrangement in six healthy subjects. The evaluation was performed during the execution of a maximum voluntary isometric contraction (MVIC) of the finger flexor muscles, using a dynamometer to measure grip strength. The R and Xc values (PhA data were not provided) increased exponentially during the exercise, regardless of whether the force was generated abruptly or gradually. Both parameters decreased once the exercise stopped, although they did not return to baseline values. Furthermore, the differences were accumulative between sets of the same exercise. The Xc values remained above basal levels after stopping the exercise, while the *R*-values were either below or above that of the resting condition. Despite no statistical analysis in the study, this technique was sensitive to physiological changes that occurred when a force was applied and to the accumulated fatigue between sets. It is worth mentioning that these results were also compared with those of subjects with neuromuscular diseases from a preliminary study [as cited in Shiffman et al. (2003)], providing different bioimpedance behaviors. Therefore, it is important to check the health of the subject before assessment.

In the same vein, Li L. et al. (2016) assessed impedance changes in 19 healthy volunteers at different levels of isometric muscle contraction until task failure (20% of the MVIC, 60% of the MVIC, and the MVIC). The muscle analyzed was the biceps brachii and the assessment was performed using an HEA multifrequency device at 50 and 100 kHz. The study also observed substantial changes during the different stages of the exercise. This time, the most significant parameter was the R, which significantly increased during exercise, especially at higher intensity contractions (60% of the MVIC and MVIC). Right after relaxing the muscle, R significantly decreased and remained above resting values (*p* = 0.014). During the post-fatigue resting period, this parameter continued to decrease (*p* = 0.011). However, this reduction was not significant compared to the resting values (*p* > 0.05). Regarding the Xc values, changes were not significant at contraction or in the resting period. There was a significant correlation between the time to muscle failure and variation in *R*-values right after relaxation (*r*^2^ = 0.3123; *p* = 0.0159). The authors suggested that the kinetics of R were due to confounding changes in architectural and metabolic factors within the contracting muscle.

Fu and Freeborn (2018) measured the biceps brachii performing an isotonic bicep curl exercise (joint angle and muscle length change while the tension remains constant) at different intensities until muscle failure. The load of the first group was set at 60% of the previously assessed one-repetition maximum (1RM), while the load of the second group was set at 75% of the 1RM. They used the tetrapolar impedance technique at 10, 50, and 100 kHz. At 50 kHz, there were significant differences in the R and Xc values (PhA data were not provided) between pre- and post-task in both groups (*p* < 0.05), but no significant differences between the two groups at different intensities, unlike the results of Li L. et al. (2016). Therefore, under the isotonic protocol applied in the study, although EIM seemed sensitive to pre-post changes, it did not seem to be able to detect differences between these intensities of exercise. However, the protocol used in the study was completely different to that in Li L. et al. (2016). Firstly, the electrode arrangement was different and, secondly, Li L. et al. (2016) used a single execution of an isometric exercise until task failure while Fu and Freeborn (2018) used multiple repetitions of an isotonic bicep curl exercise.

Freeborn and Fu (2019) analyzed 17 healthy subjects performing 10 sets of a fatiguing protocol of bicep curl repetitions until muscle failure at two different intensities: 60% of the 1RM vs. 75% of the 1RM. Impedance values were measured on the biceps brachii using the tetrapolar arrangement before the exercise and immediately after each set. Similar to the previously mentioned studies, the R, Xc, and PhA values tended to decrease at 50 kHz, with these reductions accumulating through the sets. As seen in Fu and Freeborn (2018), similar patterns were observed at both intensities, with R the first parameter to indicate a significant change (in the third set for 60% of the 1RM and in the fourth set for 75% of the 1RM). The authors suggested that R decreased due to the more localized fluid volume generated by muscle swelling, although they did not quantify it. This theory is based on the results of Yasuda et al. (2015), which showed increases in biceps brachii thickness after multiple sets of a bicep curl exercise, with muscle swelling increasing with every additional set. Furthermore, they also indicated that the Xc values may not be as affected by muscle swelling and fluid accumulation as the *R*-values and that it may serve as a marker of muscle health, fatigue, and injury. Another point to consider is that the authors did not indicate how the 1RM was assessed.

Finally, Huang et al. (2020) measured *R*-values (they did not report Xc or PhA data) at different intensities (20, 40, and 60% of the MVIC) of dynamic contractions in 8 healthy subjects. They employed a different bioimpedance device from the ones applied in the other studies, using a signal generator, an oscilloscope, a differential probe, and four electrodes linearly placed on the biceps brachii. Despite the use of a multifrequency device, the authors focused only on the outcome at 50 kHz. The study established a 3D model of the arm to optimize electrode configuration using a simulation method. Furthermore, surface electromyography was performed at the same time to verify the use of EIM as an indicator of muscle fatigue. The results showed a marked decrease at all three intensities, averaging a reduction of 7.99 ± 1.9Ω when comparing the *R*-values of fatigued vs. non-fatigued muscle. There were some consistencies when comparing R with the median frequency parameter obtained via surface electromyography. Thus, EIM can be used as an evaluator index of muscle fatigue. Ultimately, the decrease in R is affected by different load levels, declining faster as the load increases.

### Medium-Term Adaptations

Regarding the studies on medium-term adaptations, Freeborn et al. (2020) also measured localized impedance changes in the biceps brachii using a multifrequency device and arranging the four electrodes linearly over the muscle. Six subjects performed an exhausting, eccentric, bicep curl exercise (90% of the 1RM) and were measured at different time points (pre-exercise and 24, 48, 72, and 96 h post-exercise). At 50 kHz, the R and Xc values decreased from pre- to post-measurements, with the largest reductions observed at 72 h and 96 h post-exercise. The statistically significant differences in both R and Xc values occurred at the same timepoints as that of the maximum post-exercise muscle swelling, in accordance with previous studies (Li L. et al., 2016; Fu and Freeborn, 2018; Freeborn and Fu, 2019). This indicates that EIM is sensitive to the muscle swelling induced by exercise (Shiffman et al., 2003; Li L. et al., 2016; Freeborn and Fu, 2019). On the other hand, changes in Xc values were similar to those observed in severe injuries (Nescolarde et al., 2013, 2015, 2017, 2020). Information on the PhA values and how 1RM was assessed was not provided.

Elleby et al. (1990) used localized electrical impedance to assess muscle (calves and thighs) changes induced by a half marathon in four healthy subjects. Assessments were undertaken on each of the 5 days before the race, immediately after the race (2 and 6 h after the race finished), and on each of the 5 days post-race. Therefore, the analysis of exercise adaptations encompassed 6 days plus the previous 5 days. Despite the tetrapolar 50-kHz measurements, the assessment technique was different (Brown et al., 1988) and only the resistivity [real part of the complex-valued impedance per unit length and per cross-sectional area (Faes et al., 1999)] of the whole muscles was analyzed. As mentioned by the authors, the impedance of a limb is mainly resistive at 50 kHz. They measured the longitudinal and transverse resistivity of the limb, as well as the limb circumference and the hematocrit. Transverse resistivity was found to be increased immediately after the race (7%) and the values returned to baseline levels the next day. However, no changes were observed in longitudinal resistivity. Despite the fact that the limb circumference and hematocrit were consistent with dehydration and the values returned to the pre-race levels on the day following the race, the origin of the small resistivity changes was difficult to explain. It should be noted that the study presented a high risk of bias according to the AQUA tool. Therefore, their results should be interpreted with caution.

### Long-Term Adaptations

The study of Mascherini et al. (2015) was the only one analyzing long-term muscle impedance adaptations, evaluating elite soccer players during a pre-season training period of 50 days. They compared whole-body and localized (tetrapolar assessments at the quadriceps, hamstrings, and calves) BIA values. At the end of the training period, all three muscles showed significantly decreased *R*-values. The PhA increased only in the quadriceps, while the Xc decreased in the calves. Thus, the extensor muscles showed major changes. Whole-body analysis revealed an increased amount of water, especially in the lower limbs. This could explain the reduction in the R in all the muscle groups.

### Summary of Bioelectrical Adaptations to Physical Exercise

In conclusion, few studies on adaptations to exercise are currently present in the scientific literature. However, from those reviewed, we conclude that localized bioimpedance is sensitive to muscle changes occurring during exercise. Therefore, considering that more research is needed, this technique may be useful, easy to use, and a real-time tool to non-invasively quantify changes caused by exercise, muscle fatigue, and recovery.

All investigations on short-term adaptations showed a clear relationship between changes in impedance values and D-EIM during or after exercise. *R*-values appeared to be the most significant parameter, possibly because of the multiple mechanisms that take place inside the muscle as acute adaptations to exercise, for example, an increase in blood flow, vasodilation, or metabolites in the tissues (Freeborn et al., 2020). In accordance with previous studies on human subjects (Rutkove, 2009) and animal models (Sanchez et al., 2016a), *R*-values increased during contractions, as well as the Xc, and decreased after relaxation. Furthermore, this decrease accumulated over the different sets of exercise with increasing fatigue. R seemed to be the most affected, its most significant change coinciding with the time point of maximum muscle swelling. The load level also appeared to affect the behavior of the impedance values, and this is important because short-term studies did not use loads greater than 75% of 1RM, unlike medium-term studies. The similarity between the changes in Xc in a healthy muscle performing an exercise compared to an injured muscle makes it worthwhile to continue investigating this further. Regarding the PhA, there is very little information, since most studies did not report the data. Furthermore, L-BIA seemed to be appropriate in assessing hydration and cell mass in a specific body area, which may help to reduce the risk factors for injury, such as dehydration, and prevent deterioration in physical performance (Nédélec et al., 2012). One limitation of the studies looking at short-term changes was that all of them were carried out in the biceps brachii. Therefore, the results could not be extrapolated to the rest of the body given the morphological and physiological differences between the biceps brachii and the other muscles. The studies on medium-term and long-term adaptations did not provide enough information to establish relevant conclusions due to the scarcity of the investigations and the low quality of some of the studies. Furthermore, the assessment technique was not uniform across the studies, making it difficult to compare their results. Therefore, more research is needed on this.

More studies applying different training protocols and observation periods, analyzing different muscles, and using larger sample sizes are needed. Despite the fact that the present article did not aim to review the topic, it should be noted that impedance adaptations evoked by exercise are different in healthy subjects compared to people with neuromuscular diseases (Shiffman et al., 2003), which has also been reported in studies with mice (Sanchez et al., 2016a).

### The Principles of EIM

As already shown, electrical bioimpedance assessments can be performed using different types of technology and techniques and it is applicable in different contexts. This must be considered to minimize errors and obtain the most accurate and reliable measurements. Some of the factors that may affect results in localized measurements are: the anatomical characteristics of the muscle itself; the devices and materials used to measure bioimpedance; the design of the assessment technique; the characteristics and arrangement of the electrodes; the device frequency; and the preparation of the subject.

The most common way of performing EIM is using 4 electrodes linearly arranged over the muscle at a single frequency of 50 kHz. However, this was not the only protocol followed in the reviewed studies, which showed important inconsistencies even in the mentioned protocol. It is difficult to compare the results of investigations applying different techniques, considering that a small change in the measurement can significantly affect the results (Jafarpoor et al., 2013; Sanchez et al., 2016b; Rutkove et al., 2017). For this reason, it is imperative to standardize the assessment protocol. In this way, it would be interesting to develop (or even use) a methodology like the SENIAM Project, which is a surface electromyography methodology (Hermens et al., 2000) that tries to overcome the same issue, standardizing electrode placement on each muscle.

As far as material electrodes are concerned, the most common is the Ag/AgCl electrode, despite the presence of others such as dry electrodes, textile electrodes, and physiotherapy electrodes. These alternatives have their strengths and weaknesses and they can be used in specific occasions if the study design requires so (Sanchez and Rutkove, 2017a). A newer alternative is the microneedle electrode array (Li Z. et al., 2016), which has the advantage of being much more suitable for assessing small muscles and not being affected by surrounding tissues, unlike that observed with surface electrodes. However, it is more invasive than superficial electrodes.

Regarding electrode placement, an alternative used in some of the studies included in this review was an HEA. An HEA facilitates and speeds up bioimpedance assessments since the four electrodes are fixed in an array that is easy to maneuver. However, the major limitation is the close proximity of the electrodes, which may significantly affect the results because of the presence of subcutaneous tissue (Kortman et al., 2013; Sung et al., 2013; Li L. et al., 2016). An HEA is less sensitive than other methods since the electrical current penetrates only the superficial area of the muscle (Jafarpoor et al., 2013; Rutkove et al., 2017). Therefore, the great advantage of having the electrodes in a fixed position has an important drawback in that the electrode arrangement cannot be adapted to the anatomical characteristics of the muscle being assessed. This is important because the greater the separation between the electrodes, the deeper the current penetrates. Therefore, if this electrode arrangement is used, the surrounding tissues (e.g., skin, SFL, and bone) will have less impact on the assessments. However, the blood volume in the vessels and arteries may have an effect (Sanchez et al., 2016b). Hence, the big question should be where to place the different electrodes over the muscle to ensure maximum reliability. To establish the optimal electrode configuration, some studies used a finite element model based on different equations to obtain a geometrical model of the segment analyzed (Jafarpoor et al., 2013; Baidya and Ahad, 2016; Sanchez et al., 2016b; Rutkove et al., 2017), moving the electrodes to different places throughout the muscle (Jafarpoor et al., 2013; Sanchez et al., 2016b) until an appropriate location was found that improved the efficiency and fully exploited the capacity of EIM to assess the muscle. The choice of the device frequency is also important because the results may completely change. Although some of the studies used multifrequency devices, the present review only analyzed results at 50 kHz because this is the closest frequency to the one at which the Xc presents a greater value (De Lorenzo et al., 1997). However, it is also interesting to measure in the kHz-MHz range to obtain complete impedance information of the underlying structures and muscle properties. In fact, some investigations performing multifrequency analysis suggested that this may be more sensitive in the assessment of diseased muscles and their progression over time (Rutkove et al., 2012; Schwartz et al., 2015). It is also necessary to use phase-sensitive devices, since non-phase-sensitive instruments cannot measure Xc and the proper application of BIA needs the R, Xc and PhA parameters (Castizo-Olier et al., 2018). There is an advantage in using the PhA compared to the other bioelectrical parameters as it does not need to be standardized by the length of the muscle. Before measuring, some considerations must be taken into account (Castizo-Olier et al., 2018): the skin must be prepared by shaving off hair, rubbing with gel, and cleaning with alcohol; the subject must be euhydrated, with no injury or disease conditions unless that is the study objective, in a fasting state for at least 8 h, with no recent alcohol ingestion, a voided bladder, and at least 10 min of stabilization; electrode placement should be marked in longitudinal studies to preserve the same location of electrode placement and to control the temperature of the skin; the different assessments must be performed under the same conditions and environmental characteristics and, if possible, at the same time of day (to make intra-individual and inter-individual comparisons); if measurements are performed after exercise, the subject must take a shower to remove the electrolytes off the skin and reduce the skin temperature, cutaneous blood flow, and BIA parameters to basal levels; food and drink should be avoided between measurements in the evaluation of acute variations after exercise, unless that is not possible, in which case this must be registered and it should be taken into account that the ingestion may have a minimal effect if the measurement is performed <1 h after the intake; the type of exercise must be taken into consideration since post-exercise stabilization and the moment at which the measurement is performed may vary; and the menstrual cycle must be controlled for to make comparisons according to the phase.

The choice of an appropriate technique may depend on the study design, the muscle analyzed, the characteristics of the subjects, and costs. There will always be sources of error that can affect the results, although investigators must focus their efforts on minimizing these. For this reason, it is urgent to develop a common protocol for the entire scientific community interested in this topic so that EIM can be set in a more reliable scenario.

### Prospective Research Applications and Research Agenda

The application of EIM covers areas of interest other than the evaluation of neuromuscular disorders. However, EIM has not reached its full potential and, therefore, cannot be considered a fully validated instrument. For this reason, it must be further studied and improved, both at a theoretical and technological level.

Impedance values in muscles are affected by several inherent characteristics of the subjects. The clearest one is that the PhA tends to decrease with increasing age, indicating that it can be a sensitive tool for assessing muscle deterioration. Regarding gender, the reviewed articles suggested that there could be impedance differences between males and females. Furthermore, body composition may play an important role since a greater fat mass can alter bioelectrical results, especially when using certain techniques (e.g., HEA).

With regards to skeletal muscle injuries, the results are very encouraging, since a very significant relationship has been identified between injury severity and impedance values, especially for the Xc and PhA, as they are the main indicators of cell health and integrity. However, all these studies focused on the same type of subjects. Therefore, future investigations should consist of longitudinal protocols with a larger sample size, analyzing different sports, and also including female athletes.

As far as exercise is concerned, we can only make statements about short-term adaptations since the studies on medium- and long-term adaptations were scarce and of a low quality. In the studies analyzing short-term adaptations with D-EIM, the most significant parameter seemed to be R, which increased during contraction and decreased during recovery. The Xc also showed the same trends, although to a lesser extent. However, the results urge further study on the topic in order to obtain a quantitative variable to assess training load and fatigue using a non-expensive and real-time method.

As stated throughout this review, the main focus in the continued development of this method should be on standardizing a protocol that allows the assessment of any person, thereby enabling the comparison of future studies in a much more valid and reliable way. The SENIAM Project could be a protocol to follow, with the necessary modifications made to EIM.

## Limitations

The main limitations of analyzing the literature on the use of EIM in health and physical exercise were: (1) the inconsistency between the EIM assessment techniques, with different protocols and devices used; (2) the difficulty in controlling multiple sources of error that may influence the bioelectrical signal; (3) the scarcity of scientific information regarding EIM not related to the study of neuromuscular diseases; (4) the lack of analysis of some impedance parameters that could be of interest; and (5) the limited scientific evidence explaining the bioelectrical behavior of human tissues induced by exercise.

## Conclusions

The use of EIM has grown in recent years, along with the quality of the studies. The increased interest in this technique comes mainly from the clinical field, although it may be useful in other contexts, as shown in the present review. EIM is easy to use and quick to perform, which can be applied at any time and in any place. Furthermore, it is not expensive and can be applied to a great variety of superficial muscles. Although not yet fully defined, EIM is sensitive to the muscular adaptations produced by physical exercise and sports. Therefore, it could become a great tool to control the load in athletes, as well as in the recovery process after a muscle injury. However, more research is needed on this and for EIM to be applied as a tool with a greater scientific use given that EIM has some advantages that other methods lack. For this, the first step must be the standardization of an appropriate protocol.

## Data Availability Statement

The raw data supporting the conclusions of this article will be made available by the authors, without undue reservation.

## Author Contributions

ÁC-P, AI, JC-O, and GS-L: conceptualization. ÁC-P and MC-M: data curation. ÁC-P, AI, and MG-F: formal analysis. ÁC-P, AI, and MC-M: investigation. ÁC-P, AI, GS-L, and MG-F: methodology. ÁC-P: project administration and writing and original draft. AI, JC-O, and GS-L: supervision. ÁC-P, AI, JC-O, and MC-M: writing and review & editing. All authors contributed to the article and approved the submitted version.

## Funding

ÁC-P was a pre-doctoral researcher supported by a grant within the field of physical education, physical activity, and sports and its applied sciences given to the National Institute of Physical Education of Catalonia (INEFC), University of Barcelona (UB) (2019 PINEFC 00001). The funders had no role in the study design, data collection and analysis, decision to publish, or preparation of the manuscript.

## Conflict of Interest

The authors declare that the research was conducted in the absence of any commercial or financial relationships that could be construed as a potential conflict of interest.

## Publisher's Note

All claims expressed in this article are solely those of the authors and do not necessarily represent those of their affiliated organizations, or those of the publisher, the editors and the reviewers. Any product that may be evaluated in this article, or claim that may be made by its manufacturer, is not guaranteed or endorsed by the publisher.

## Abbreviations

BIA: bioelectrical impedance analysis
BIVA: bioelectrical impedance vector analysis
D-EIM: dynamic electrical impedance myography
EIM: electrical impedance myography
HEA: handheld electrode array
L-BIA: localized bioelectrical impedance analysis
MRI: magnetic resonance imaging
MVIC: maximum voluntary isometric contraction
PhA: phase angle
R: bioelectrical resistance
RTP: return to play
SFL: subcutaneous fat layer
Xc: bioelectrical reactance
1RM: one-repetition maximum.
